# The Impact of Idiopathic Pulmonary Fibrosis on the Incidence and Severity of Radiation Pneumonitis in Non-Small Cell Lung Cancer Patients Following Radiotherapy

**DOI:** 10.3390/biomedicines14102280

**Published:** 2026-10-08

**Authors:** Byung Wook Oh, Hye Min Kim, Geun In Lee, Seung Hyun Yong, A La Woo, Eun Young Kim, Sang Hoon Lee

**Affiliations:** 1 Department of Internal Medicine, Division of Pulmonology and Critical Care Medicine, Severance Hospital, Yonsei University College of Medicine, Seoul 03722, Republic of Korea; byungwookoh@yuhs.ac (B.W.O.); hmkim20@yuhs.ac (H.M.K.); nerrnerr86@yuhs.ac (G.I.L.); roneirire@yuhs.ac (S.H.Y.); alwoo@yuhs.ac (A.L.W.); narae97@yuhs.ac (E.Y.K.); 2 Department of Medicine, College of Medicine, Inje University, Gimhae 50834, Republic of Korea

**Keywords:** non-small cell lung cancer, idiopathic pulmonary fibrosis, radiation pneumonitis, radiotherapy

## Abstract

**Background/Objectives**: Idiopathic pulmonary fibrosis (IPF) is known to increase the risk of severe pulmonary toxicity in non-small cell lung cancer (NSCLC) patients undergoing radiotherapy. This study aimed to evaluate the impact of comorbid IPF on the incidence and severity of radiation pneumonitis (RP) and overall survival (OS). **Methods**: This retrospective cohort study included NSCLC patients who received radiotherapy to the lung between 2015 and 2025. Patients were stratified into an IPF-comorbid group and a non-IPF control group. To minimize selection bias, variable-ratio (up to 1:2) propensity score matching with a caliper of 0.2 SD and survey-weighted analyses were utilized. **Results**: After matching, 196 patients (72 with IPF, 124 without IPF) were analyzed. The IPF-comorbid group had a significantly higher risk of any-grade RP (OR = 2.75, *p* = 0.001) and grade ≥ 2 RP (OR = 2.87, *p* = 0.002). In multivariable analysis, comorbid IPF was independently associated with any-grade RP (adjusted OR = 3.55, *p* = 0.001) and grade ≥ 2 RP (adjusted OR = 3.79, *p* = 0.001). With death as a competing event, the 6-month cumulative incidence of RP was 51.8% vs. 25.2%. IPF was independently associated with worse OS (adjusted HR = 2.51, *p* = 0.006), with a lower 3-year OS rate (44.4% vs. 68.1%). **Conclusions**: Comorbid IPF was independently associated with an increased risk of RP and overall mortality in NSCLC patients receiving radiotherapy. Because the cumulative incidence of RP diverged within the first months after radiotherapy, close multidisciplinary monitoring is warranted during this early period.

## 1. Introduction

Idiopathic pulmonary fibrosis (IPF) is a disease of unknown etiology that causes chronic fibrosis of the pulmonary interstitium and presents with characteristic radiologic and histologic findings [1]. It is characterized by progressive dyspnea and dry cough and carries a very poor prognosis. One factor that further worsens the prognosis of IPF is its high rate of comorbid lung cancer. According to Korean nationwide data, the incidence of lung cancer among IPF patients has been reported at approximately 22.9 per 10,000 person-years, roughly five times higher than in the general population, and the 10-year cumulative incidence of lung cancer among IPF patients reaches 31.1% [2].

Lung cancer is broadly classified into two main histological subtypes: non-small cell lung cancer (NSCLC) and small cell lung cancer (SCLC). NSCLC accounts for approximately 85% of all lung cancer cases. NSCLC is a pathological classification encompassing adenocarcinoma (adenoca), squamous cell carcinoma (SCC) and large cell carcinoma (LCC), and other less common subtypes [3]. And treatment strategy—surgical resection, radiotherapy (RT) alone, concurrent chemoradiotherapy (CCRT), or chemotherapy—varies according to stage. Patients with comorbid IPF are known to be at increased risk of acute exacerbation and pneumonitis during treatment [4,5,6,7,8].

Early-stage NSCLC without lymph node metastasis (N0, stage I–II) is generally treated with surgical resection; however, IPF patients frequently undergo stereotactic body radiotherapy (SBRT) because of impaired pulmonary function [9]. Nevertheless, the risk of radiation pneumonitis (RP) after SBRT is well recognized, and comorbid interstitial pneumonia has been reported as a risk factor for RP after SBRT in early-stage NSCLC [10,11,12,13,14]. In one study, the overall incidence of extended RP after RT was only 1.4%, yet patients with comorbid interstitial lung disease (ILD)—who comprised only 13% of the entire cohort—accounted for approximately 78% of these cases. Multivariable logistic regression analysis likewise showed a significantly higher risk of RP in patients with underlying ILD (odds ratio = 22.6, 95% CI 5.29–155, *p* < 0.001) [15]. In a study by Glick et al., the incidence of RP after SBRT was also significantly higher in the ILD group than in the non-ILD group (grade ≥ 2: 20.5% vs. 5.8%, *p* < 0.01; grade ≥ 3: 10.3% vs. 1.0%, *p* < 0.01) [13].

In stage III patients receiving CCRT, even subclinical ILD has been reported as a risk factor for RP incidence [16]. Among chemotherapeutic agents, gemcitabine, in particular, is highly associated with the development of RP. For instance, compared to docetaxel induction chemotherapy, gemcitabine was associated with a higher incidence of grade ≥ 2 RP [17]. Meanwhile, in stage IV lung cancer, although the incidence of acute exacerbation was higher with chemotherapy than with conservative management, mortality was lower, suggesting that active treatment is warranted even in lung cancer patients with comorbid IPF [18].

Although an association between pre-existing ILD and RP is well established [4,10,11,12,13,14,15], most previous studies included heterogeneous ILD or interstitial lung abnormality (ILA) populations, compared groups that differed substantially at baseline, and seldom examined RP and survival in the same cohort. Therefore, this study aimed to evaluate whether the incidence and severity of RP differ according to the presence of comorbid IPF in patients with NSCLC, using a propensity score matching (PSM) analysis to minimize baseline confounding factors. We additionally examined overall survival in the same cohort.

## 2. Materials and Methods

### 2.1. Subjects

This study included patients diagnosed with lung cancer between 1 January 2015 and 31 December 2025, identified through the Severance Clinical Research Analysis Portal (SCRAP). The inclusion criteria were as follows: (1) adults aged ≥19 years; (2) patients with histologically confirmed NSCLC or clinically diagnosed lung cancer via multidisciplinary consensus; (3) patients who received RT directly to the lung; and (4) patients who received their first RT for lung cancer between 1 January 2015 and 31 December 2025. The exclusion criteria were as follows: (1) patients diagnosed with another primary cancer within 5 years of lung cancer diagnosis; (2) patients who did not receive RT directly to the lung; (3) patients with no clinical or radiological assessment after RT; (4) patients whose treatment was discontinued during RT due to deterioration of general condition; and (5) patients treated with RT for lung cancer before 2015.

This retrospective study was approved by the Institutional Review Board (IRB) of Severance Hospital, Yonsei University Health System (IRB No. 4-2026-0416). The requirement for obtaining informed patient consent was waived by the IRB due to the retrospective nature of this study and the use of anonymized clinical data. All study procedures were conducted in accordance with the ethical principles of the Declaration of Helsinki.

### 2.2. Methods

Patients’ demographic characteristics and imaging findings were confirmed through a retrospective review of the Severance electronic medical record (EMR). Based on the 2018 ATS/ERS/JRS/ALAT criteria, the experimental group was defined as patients with either a radiologic UIP or probable UIP pattern or a pathological diagnosis of IPF, and the control group was defined as patients without any imaging findings of ILD or ILA [19]. Patients with any documented radiotherapy were included in the analysis. The classification was applied retrospectively by the investigators on the basis of chest computed tomography (CT) reports and clinical records, and all patients in the IPF group had been evaluated by the pulmonology division. Known causes of a UIP pattern were excluded during pulmonology evaluation, including autoimmune serology with rheumatology referral where indicated, and patients diagnosed with connective tissue disease-associated ILD (CTD-ILD), non-specific interstitial pneumonia (NSIP) or smoking-related ILD were excluded.

**Figure 1:**
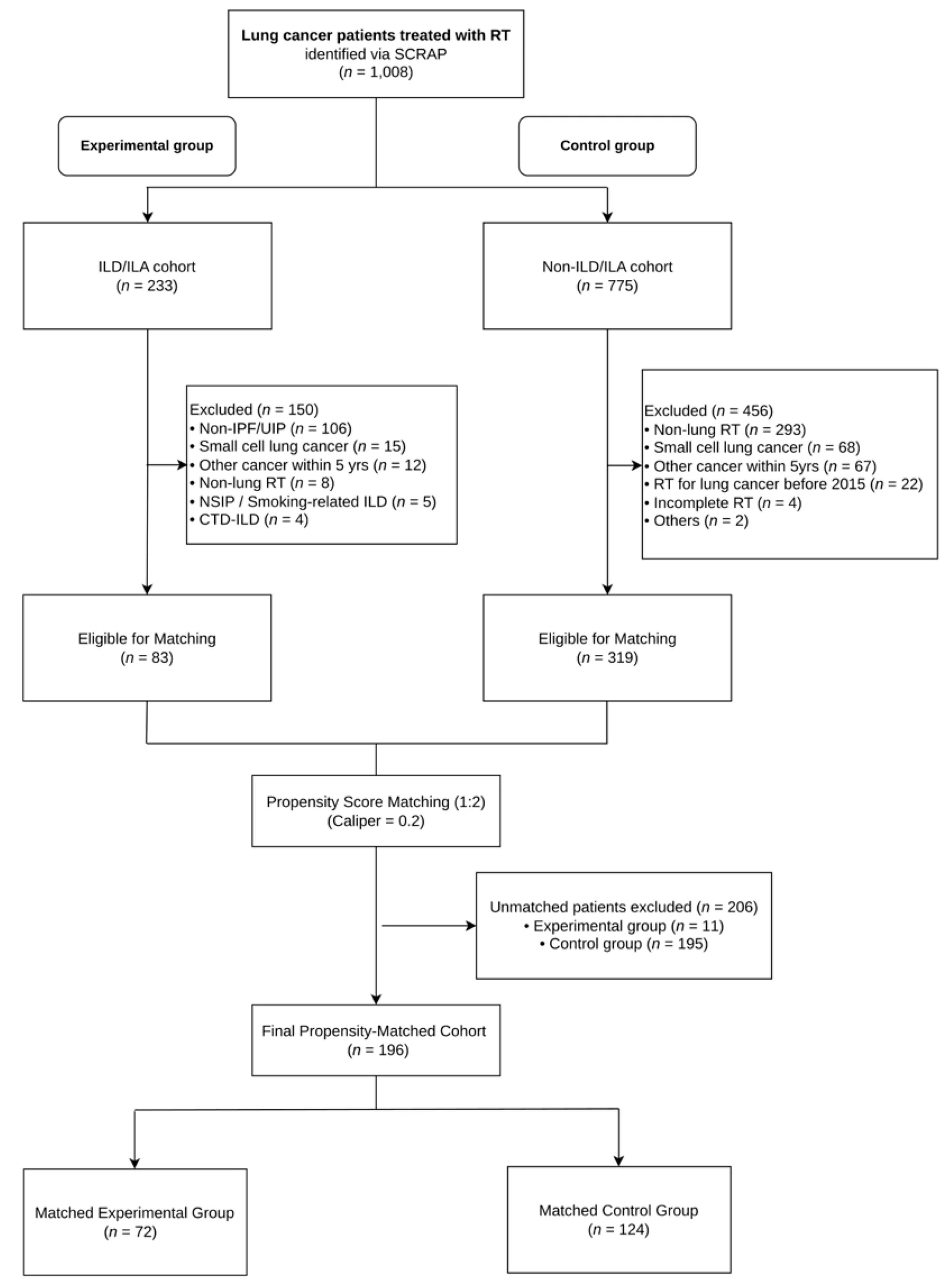
Patient flow chart of study inclusion. RT, radiotherapy; ILD, interstitial lung disease; ILA, interstitial lung abnormality; IPF, idiopathic pulmonary fibrosis; NSIP, non-specific interstitial pneumonia; CTD, connective tissue disease.

Patients diagnosed with lung cancer between 1 January 2015 and 31 December 2025, who had documented chest CT and RT records, were identified using the SCRAP. Patients with diagnosis codes for other malignancies, such as esophageal, stomach, or laryngeal cancers, were excluded. Through this process, a total of 1008 patients were identified (Figure 1). Among them, patients with imaging findings of “UIP,” “usual interstitial pneumonia;” “ILD,” “interstitial lung disease;” “NSIP;” or “ILA” were classified into the ILD/ILA cohort, while the remaining patients were assigned to the control group. From this ILD/ILA cohort, a total of 150 patients were further excluded: 106 patients without UIP (non-IPF/UIP), 15 patients with SCLC, 12 patients diagnosed with another primary cancer within 5 years, 8 patients who received RT to a site other than the lung, 5 patients ultimately diagnosed with NSIP or smoking-related ILD after pulmonology evaluation, and 4 patients diagnosed with CTD-ILD after testing positive for autoimmune antibodies and rheumatology evaluation—resulting in a final experimental group of 83 patients. On chest CT, 58 patients (80.6%) in the IPF group had a UIP pattern, and 14 (19.4%) a probable UIP pattern; no patient had an indeterminate or alternative-diagnosis pattern. Histopathological material was available in 9 patients (12.5%), showing a UIP pattern in 7, a probable UIP pattern in 1 and an alternative pattern in 1.

The control group was obtained by excluding patients with imaging findings of ILD/ILA, yielding an initial list of 775 patients. Of these, 456 patients were excluded: 293 who received RT to sites other than the lung (e.g., spine, brain), 68 with SCLC, 67 diagnosed with another primary cancer within 5 years, 22 who received RT for lung cancer before 2015, 4 whose treatment was discontinued during RT, and 2 for other reasons—resulting in a final control group of 319 patients.

No a priori sample size calculation was performed; the study size was determined by the total number of consecutive patients who met the eligibility criteria and received thoracic radiotherapy at our institution during the 11-year study period, and the final analytical sample was defined by the propensity score matching procedure.

Radiotherapy details, including total dose, dose per fraction and number of fractions, were abstracted from treatment records, and regimens were classified by dose per fraction (≤2.5, >2.5 to <6 and ≥6 Gy per fraction), an objective and reproducible criterion that distinguishes conventionally fractionated from stereotactic regimens. The diagnosis of RP was retrospectively determined based on a combination of clinical symptoms and radiologic findings. RP was defined by radiographic changes (such as ground-glass opacities or consolidation) corresponding to the radiation field following the completion of RT, with or without accompanying respiratory symptoms [20]. Opacities extending beyond the radiation field, or with a distribution inconsistent with the treated volume, were not classified as RP. Acute exacerbation of IPF, characterized by new bilateral infiltration superimposed on the background fibrotic pattern and not confined to the radiation field, was recorded separately; it occurred in 14 of the 72 patients with IPF (19.4%) during follow-up. Immune checkpoint inhibitor (ICI)-related pneumonitis was distinguished by its distribution beyond the treated field and its temporal relationship to immunotherapy, and ICI exposure was included as a covariate in the multivariable analysis. Tumor progression was distinguished by mass-like morphology on serial imaging. Infection within a previously irradiated field cannot be reliably distinguished from RP on imaging; the two were therefore not treated as mutually exclusive, and most patients with suspected RP accompanied by clinical features of infection received empirical antibiotics concurrently with corticosteroids. Cases with diagnostic uncertainty were reviewed and resolved by a multidisciplinary team. RP was graded according to the Common Terminology Criteria for Adverse Events (CTCAE) v6.0 (MedDRA 28.0), based on the change from each patient’s pre-radiotherapy baseline. Grade 1 was defined as asymptomatic; Grade 2, symptoms affecting quality of life; Grade 3, requiring new or increased supplemental oxygen; Grade 4, requiring mechanical ventilation or non-invasive ventilation; and Grade 5, death.

### 2.3. Statistical Analysis

To balance baseline characteristics between the experimental and control groups, PSM was performed by nearest-neighbor matching without replacement on a propensity score estimated by logistic regression, with a caliper of 0.2 standard deviations of the propensity score. The variables included in matching were sex, body mass index (BMI), smoking history, ECOG performance status (PS), histologic confirmation status, TNM stage, predicted forced vital capacity (FVC), intent of RT (palliative vs. definitive), and total radiation dose. To strictly adhere to the caliper criterion and ensure high-quality matches, a variable matching ratio (up to 1:2) was applied. Matching weights were therefore calculated to correct for the resulting imbalance according to the number of matched controls and to accurately estimate the average treatment effect on the treated. All subsequent statistical analyses, including comparisons of RP incidence and survival, were performed using models incorporating these weights (survey-weighted analysis). After matching, a total of 196 patients were included in the analysis: 72 in the experimental group and 124 in the control group (Figure S1). Matching was performed with the MatchIt package in R; 52 patients in the experimental group were matched to two controls and 20 to one control, and 11 without a control within the caliper were not matched (Figure 1). The effective sample size after weighting was 184.3 (72.0 in the experimental group and 112.7 in the control group).

Percent-predicted FVC, a matching covariate, was missing in 24 of the 196 matched patients (12.2%; IPF 5, non-IPF 19), in whom forced expiratory volume in 1 s (FEV_1_) and FEV_1_/FVC were also unavailable, and the diffusing capacity of the lung for carbon monoxide (DLco) was missing in 62 patients (31.6%; 14 and 48, respectively); all other variables were complete. Missing FVC was imputed under a missing-at-random assumption by multiple imputation by chained equations with predictive mean matching (MICE-PMM) in the full pre-matching cohort, before propensity score estimation. The imputation model included IPF status, all propensity score covariates, smoking pack-years and pathological type but no outcome variables, so imputation and matching were performed without reference to outcomes; five imputed datasets were generated (mice package in R, fixed random seed), and the first completed dataset was used for PSM and all subsequent analyses (single imputation; estimates were not pooled across imputations). The target estimand was the average effect of comorbid IPF in patients with IPF (analogous to the average treatment effect on the treated), relative to non-IPF patients with comparable clinical characteristics, radiotherapy and baseline lung volume; because FVC may itself be reduced by IPF, the estimate represents the effect of IPF among patients with comparable FVC rather than its total effect. DLco was not a matching covariate because of its high and differential missingness and because a reduced DLco with a preserved FEV_1_/FVC ratio is characteristic of IPF and would have limited the overlap between groups; FEV_1_ was excluded because it was strongly correlated with FVC (r = 0.63). These three indices were analyzed as observed, and their influence was examined in sensitivity analyses. After applying propensity score weighting, differences in baseline clinical characteristics between the two groups were compared using the Rao–Scott chi-square test for categorical variables and the survey-weighted Wald test for continuous variables. Standardized mean differences (SMDs) were calculated to assess covariate balance after weighting, with an SMD < 0.1 considered indicative of adequate balance. To account for weighting imbalance, differences in RP incidence between groups were assessed using the Rao–Scott chi-square test, and odds ratios (ORs) were calculated using survey-weighted logistic regression. Time to onset of RP from the start of RT was compared among patients who developed RP using the Wilcoxon rank-sum test and was interpreted descriptively, because this comparison is conditional on the occurrence of RP. Survey-weighted logistic regression was also used for multivariable analyses of any-grade, grade ≥ 2 and grade ≥ 3 RP; because these models were fitted in the matched and weighted sample, they additionally adjusted for factors that were not matching covariates. Candidate covariates were screened by univariable analysis (*p* < 0.2), with age and sex forced into the models. Due to the small number of events, the model for grade ≥ 3 RP was adjusted for age and sex only (19 events).

In sensitivity analyses for any-grade RP, total radiation dose was replaced by the biologically effective dose (BED_3_, α/β = 3 Gy) or by fractionation category; tumor location was added to the multivariable model; the analysis was restricted to patients treated with ≥6 Gy per fraction or to patients with a definite UIP pattern and their matched controls; and DLco or FEV1/FVC was added among patients with measured values. The analysis was repeated among the 172 patients in whom FVC had been measured (complete-case analysis). The cumulative incidence of RP from the start of RT was estimated with death without RP as a competing event (weighted Aalen–Johansen estimator), and the effect of IPF with Fine–Gray subdistribution and cause-specific hazard models with robust variance. In an exploratory analysis among patients with IPF, RP incidence was compared according to antifibrotic use using Fisher’s exact test.

Weighted Cox proportional hazards regression was used to assess survival according to IPF status, and survey-weighted Cox proportional hazards regression incorporating multivariable factors was used for adjusted survival analysis. Weighted Kaplan–Meier curves were used to compare survival between groups. Overall survival (OS) was measured from lung cancer diagnosis to death from any cause, with censoring at the last follow-up, and the median follow-up was estimated by the reverse Kaplan–Meier method. Where the proportional hazards assumption, assessed with scaled Schoenfeld residuals, was not satisfied, a model allowing the effect of IPF to differ before and after 24 months and the weighted restricted mean survival time up to 36 months (bootstrap 95% CI) were also examined. Statistical analyses were performed using RStudio (version 2026.7.0.139).

## 3. Results

### 3.1. Baseline Characteristics

Patient characteristics are listed in Table 1. Among demographic characteristics, age (SMD = 0.011), sex (SMD = 0.023), and BMI (SMD = 0.012) showed no significant difference between the two groups, with SMD values ≤ 0.1. Regarding underlying conditions and smoking history, smoking status (SMD = 0.015) and asthma (SMD = 0.109) likewise showed no between-group difference. Among treatment-related characteristics, there were no significant differences in the intent of RT (SMD = 0.064) or total radiation dose (SMD = 0.054). Regarding RT fractionation, ≥6 Gy per fraction was used in 76.4% vs. 70.1%, >2.5 to <6 Gy in 6.9% vs. 9.0% and ≤2.5 Gy in 16.7% vs. 20.8% of the IPF-comorbid and non-IPF groups, respectively (SMD = 0.142); BED_3_ was well balanced (SMD = 0.047), whereas the SMDs for dose per fraction (0.177) and number of fractions (0.126) slightly exceeded 0.1.

In contrast, prior chemotherapy (SMD = 0.443), the prior use of ICI (SMD = 0.430), prior extrapulmonary malignancy (SMD = 0.418), and hypertension (SMD = 0.309) showed statistically significant differences between the two groups. These variables were not included in the propensity score model; of these, ICI exposure was adjusted for in the multivariable analyses.

The pulmonary function test results also showed marked differences, with SMD values of approximately 0.5–0.8 or greater for predicted FEV1, FEV1/FVC, and DL_CO_. Predicted FEV1 and FEV1/FVC were higher in the IPF-comorbid group, whereas DL_CO_ was markedly lower.

Among patients in the IPF group, approximately 58.3% received ILD-directed treatment; 51.4% received antifibrotic agents and 6.9% received immunosuppressants including corticosteroids.

### 3.2. Radiation Pneumonitis

In the matched cohort, RP occurred more frequently and with greater severity in the IPF-comorbid group compared to the non-IPF group. The incidence of any-grade RP was significantly higher in the IPF-comorbid group than in the non-IPF group (55.6% [40/72] vs. 30.6% [38/124]; *p* = 0.001). Similarly, the IPF-comorbid group exhibited significantly higher rates of grade ≥ 2 RP (43.1% vs. 20.2%; *p* = 0.002) and grade ≥ 3 RP (16.7% vs. 5.6%; *p* = 0.040) (Table 2). Fatal grade 5 RP was observed in two patients (2.8%) in the IPF-comorbid group, whereas no such events occurred in the non-IPF group. Among patients who developed RP, the median time to onset was shorter in the IPF-comorbid group (111.5 days, IQR 62.0–145.5) compared to the non-IPF group (126.5 days, IQR 84.8–150.8); however, this difference was not statistically significant (*p* = 0.060). The risk of RP was further analyzed using propensity score-weighted logistic regression. The IPF-comorbid group showed a statistically significantly higher risk of RP than the non-IPF group across all severity grades (Table 2). The risk of any-grade RP was significantly increased in the IPF-comorbid group (OR = 2.75, 95% CI 1.48–5.11, *p* = 0.001). The risk of grade ≥ 2 RP, reflecting clinically significant symptoms, was approximately 2.87-fold higher in the IPF-comorbid group (OR = 2.87, 95% CI 1.48–5.57, *p* = 0.002). The risk of grade ≥ 3 RP, classified as severe, was also significantly higher in the IPF-comorbid group (OR = 3.00, 95% CI 1.07–8.38, *p* = 0.036).

Additionally, univariate propensity score-weighted logistic regression was performed to evaluate other potential risk factors for RP (Table 3). The evaluated clinical and treatment-related variables included age, gender, ECOG PS, smoking history, smoking pack-years, prior extrapulmonary malignancy, pathology type, prior lung surgery history, exposure to ICI, receipt of CCRT, tumor size, predicted FVC, and total RT dose. In the univariate analysis, the receipt of CCRT (OR = 2.38, 95% CI: 1.17–4.84, *p* = 0.017) and larger tumor size (OR = 1.40, 95% CI: 1.04–1.88, *p* = 0.025) were significantly associated with an increased risk of RP.

For the multivariable propensity score-weighted logistic regression, all variables demonstrating a potential association (*p* < 0.20) in the univariate analysis were included, with age and gender forced into the model. Consequently, the final adjusted model included comorbid IPF, age, gender, prior lung surgery history, ICI exposure, CCRT receipt, tumor size, and total RT dose. In this multivariable model, comorbid IPF remained a strong, independent risk factor for RP (adjusted OR [aOR] = 3.55, 95% CI: 1.74–7.24, *p* = 0.001). Furthermore, increasing age (aOR = 1.04, 95% CI: 1.00–1.08, *p* = 0.027), prior lung surgery history (aOR = 3.14, 95% CI: 1.13–8.71, *p* = 0.029) and increasing total radiation dose (aOR = 1.03 per 1 Gy increase, 95% CI: 1.00–1.07, *p* = 0.041) were significantly associated with an elevated risk of RP. However, factors such as CCRT and tumor size, which were significant in the unadjusted analysis, did not retain their statistical significance after adjusting for other confounders (Table 3). The same covariate set was applied to the severity-specific endpoints: the association persisted for grade ≥ 2 RP (aOR = 3.79, 95% CI 1.77–8.09, *p* = 0.001) and, adjusted for age and sex only because of the small number of events (*n* = 19), for grade ≥ 3 RP (aOR = 3.03, 95% CI 1.07–8.60, *p* = 0.037) (Table S2).

The association between comorbid IPF and RP was consistent in all sensitivity analyses (Table S2; adjusted ORs for any-grade RP 3.32–5.71), including when total dose was replaced by BED_3_ or fractionation category, when the analysis was restricted to patients treated with ≥6 Gy per fraction or to patients with a definite UIP pattern and their matched controls, when DLco or FEV1/FVC was added among patients with measured values, and when the analysis was restricted to the 172 patients in whom FVC had been measured (complete-case analysis). BED_3_ itself was not associated with RP (*p* = 0.674), whereas regimens of ≥6 Gy per fraction were associated with a lower risk of RP than regimens of ≤ 2.5 Gy per fraction (33.1% vs. 61.1%; adjusted OR 0.12, 95% CI 0.02–0.59; Table S3). Tumor location was not significantly associated with RP, and the association of comorbid IPF with RP was maintained after adjustment for it (Tables S2 and S3).

With death without RP as a competing event, the cumulative incidence of RP in the IPF-comorbid and non-IPF groups was 25.2% vs. 9.7% at 3 months, 51.8% vs. 25.2% at 6 months and 56.6% vs. 32.0% at 12 months, with little further increase thereafter (Figure S2); the subdistribution and cause-specific hazard ratios for comorbid IPF were 2.36 (95% CI 1.50–3.72) and 2.38 (95% CI 1.51–3.75), and 2.46 and 2.60 after adjustment for the covariates of the primary model (all *p* < 0.001; Table S2).

An additional analysis was performed among matched patients with IPF to evaluate the effect of antifibrotic use on RP incidence. RP incidence was numerically lower among patients receiving antifibrotics (51.4%; 19 of 37) than among those not receiving antifibrotics (60.0%; 21 of 35). However, this difference was not statistically significant (Fisher’s exact test, *p* = 0.487). The OR for RP according to antifibrotic use was 0.71 (95% CI 0.25–1.98).

### 3.3. Overall Survival

The median follow-up (reverse Kaplan–Meier method) was 27.0 months (IQR 15.0–48.0) overall, 35.0 months in the IPF-comorbid group and 26.0 months in the non-IPF group (417 person-years). A total of 30 of 72 patients (41.7%) in the IPF-comorbid group and 31 of 124 (25.0%) in the non-IPF group died, and 27 (17 and 10, respectively) were censored after loss to follow-up. Causes of death were pneumonia (*n* = 11), acute exacerbation of IPF (*n* = 3), RP (*n* = 2), cardiac disease (*n* = 1) and other causes (*n* = 13) in the IPF-comorbid group and pneumonia (*n* = 8), other infection or sepsis (*n* = 1) and other causes (*n* = 22) in the non-IPF group.

To identify prognostic factors for OS, univariable and multivariable propensity score-weighted Cox proportional hazards regression analyses were performed (Table 4). In the univariable analysis, the presence of IPF (hazard ratio [HR] = 1.87, 95% CI: 1.12–3.14, *p* = 0.018), poorer ECOG PS (HR = 1.49, *p* = 0.009), higher smoking pack-years (HR = 1.01, *p* = 0.026), specific pathology types (all *p* < 0.001), receipt of CCRT (HR = 2.60, *p* < 0.001) and higher TNM staging (HR = 1.66, *p* < 0.001) were significantly associated with an increased risk of mortality. Conversely, higher predicted FVC was associated with a decreased risk of mortality (HR = 0.97, *p* = 0.003).

Variables with a *p*-value < 0.20 in the univariable analysis, along with age and gender, were included in the multivariable adjusted model. After adjusting for potential confounders, the presence of IPF remained a strong and independent predictor of worse OS (adjusted HR [aHR] = 2.51, 95% CI: 1.30–4.84, *p* = 0.006). Furthermore, advanced TNM staging (aHR = 1.51, 95% CI: 1.08–2.11, *p* = 0.016) and specific pathology types (adenoca: *p* = 0.011; others: *p* < 0.001) retained their significance as independent poor prognostic factors. Higher predicted FVC remained an independent protective factor for OS (aHR = 0.97, 95% CI: 0.95–0.99, *p* = 0.015).

**Figure 2:**
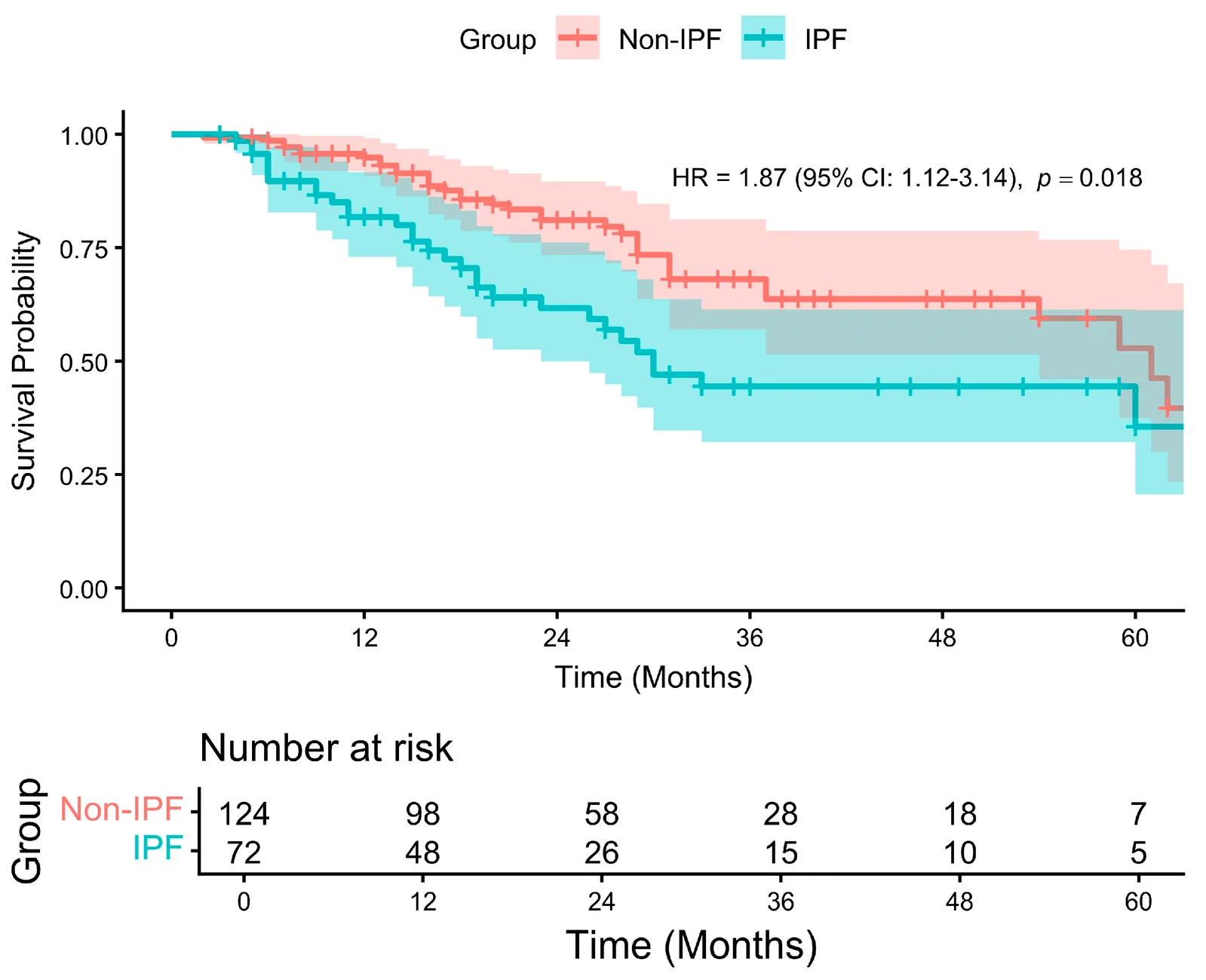
Weighted Kaplan–Meier survival curve.

Weighted Kaplan–Meier analysis was used to evaluate OS over time (Figure 2). Throughout the follow-up period, survival in the IPF-comorbid group remained consistently lower than in the non-IPF group (*p* = 0.011; Table S1). The 3-year OS rates were 44.4% and 68.1% in the IPF-comorbid and non-IPF groups, respectively.

The proportional hazards assumption was not satisfied for IPF status (*p* = 0.002; global test, *p* = 0.010): the adjusted HR for IPF was 3.98 (95% CI 1.76–9.02, *p* = 0.001) during the first 24 months after diagnosis and 1.14 (95% CI 0.40–3.22, *p* = 0.806) thereafter, so the aHR of 2.51 represents a time-averaged estimate. The weighted restricted mean survival time up to 36 months was 25.9 months in the IPF-comorbid group and 31.2 months in the non-IPF group (difference −5.2 months, 95% CI −8.6 to −1.8).

## 4. Discussion

This study demonstrated that among NSCLC patients undergoing RT, those with comorbid IPF had a significantly increased risk of RP across all severity grades, with a cumulative incidence that diverged early after RT. In multivariable analysis, comorbid IPF was independently associated with both the development of RP (aOR = 3.55) and reduced overall survival (aHR = 2.51). In addition, advanced TNM stage and lower baseline predicted FVC were identified as major factors threatening patient survival. Although an association between ILD and RP has been reported previously, our findings add IPF-specific estimates from a propensity score-matched cohort in which RP, its severity and survival were assessed together, and the association persisted after accounting for competing mortality, fractionation and baseline lung function.

In our study, the incidence of RP was significantly higher in patients with IPF compared to those without IPF (grade ≥ 2: 43.1% vs. 20.2%; grade ≥ 3: 16.7% vs. 5.6%). This aligns with a prior study comparing similar cohorts, which demonstrated rates of 20.5% vs. 5.8% for grade ≥ 2 RP and 10.3% vs. 1.0% for grade ≥ 3 RP [13]. Furthermore, a recent systematic review reported an overall grade 3 RP incidence of 19.7% (range 8–46%), closely matching our finding of 16.7% [12]. Our analysis identified comorbid IPF (aOR = 3.55), increasing age (aOR = 1.04), prior lung surgery history (aOR = 3.14), and increasing total radiation dose (aOR = 1.03) as independent risk factors for RP. However, the association with total dose was not reproduced with BED_3_ or fractionation category, suggesting that it reflected differences in fractionation rather than a dose–response relationship; total prescription dose should therefore not be regarded as a modifiable predictor. The increased risk associated with prior lung surgery may be attributed to a reduction in total lung volume; if the absolute radiation dose is not proportionally reduced, the dose delivered to the remaining normal lung tissue inherently increases. Consistent with our findings, a previous study reported age and larger tumor size as significant predictors of RP [21]. While tumor size did not retain significance in our multivariable model, it did show a similar significant trend in our univariate analysis. Another study identified UIP pattern (OR = 5.49), diabetes mellitus, CCRT and ICI as significant factors [20]. In our cohort, CCRT was significantly associated with RP in the univariate analysis.

Age was associated with RP only after adjustment (OR = 1.01, *p* = 0.668; aOR = 1.04, *p* = 0.027), consistent with negative confounding: older patients more often received regimens of ≥6 Gy per fraction (OR 1.09 per year, *p* < 0.001), which are generally delivered to small target volumes; less often received CCRT (OR 0.90 per year, *p* < 0.001) or ICI; and had smaller tumors. These factors would mask an effect of age in the unadjusted analysis.

Regarding overall survival, comorbid IPF (aHR = 2.51), advanced TNM staging (aHR = 1.51), and specific pathological types were significantly associated with increased mortality risk. However, the survival difference cannot be attributed specifically to RT toxicity: RP accounted for only 2 of the 30 deaths in the IPF-comorbid group, whereas pneumonia and acute exacerbation of IPF accounted for 14, consistent with the substantial mortality inherent to IPF. Moreover, the excess hazard was concentrated within the first 24 months after diagnosis. The higher aHR associated with advanced TNM staging reflects the prognostic discrimination of the 9th edition TNM staging system [22]. Interestingly, patients who underwent pathological diagnosis exhibited a relatively higher aHR. This counterintuitive result is likely due to the unexpectedly favorable survival of the unbiopsied group. In patients with IPF, biopsies are frequently omitted due to the high risk of procedure-related complications. More importantly, in clinical practice, biopsies are often waived for patients with small, early-stage peripheral tumors that are technically difficult to access safely. As a result, these early-stage lesions are highly amenable to empirical radiotherapy and inherently carry a better prognosis.

While recent evidence suggests that antifibrotic agents can reduce the incidence of acute exacerbation following surgical resection or after the onset of RP, data regarding their efficacy in preventing RP remains scarce [23]. In our study, the use of antifibrotic agents did not demonstrate a statistically significant protective effect against RP. This exploratory, hypothesis-generating comparison was non-randomized and susceptible to confounding factors like differences in IPF severity, immortal time bias, timing and duration of therapy. Therefore, further large-scale prospective studies are warranted to definitively clarify their preventive role.

A strength of this study is the use of propensity score matching, survey-weighted analyses, competing-risk methods and multiple sensitivity analyses, in contrast to many previous observational studies in which baseline characteristics differed substantially between groups. Beyond a simple comparison of RP incidence, our study provides adjusted real-world risk estimates for comorbid IPF, older age and prior lung surgery, which can inform patient selection, informed consent and post-radiotherapy monitoring. Nevertheless, residual imbalance and unmeasured confounding remain and should be considered when interpreting the adjusted associations.

However, this study has several limitations. First, approximately 40% of the patients in our matched cohort lacked histological confirmation of NSCLC. Given the high risk of complications in patients with IPF, biopsies were frequently omitted. Consequently, we cannot entirely rule out the unintended inclusion of SCLC. Nevertheless, we consider this probability to be low. Epidemiologically, SCLC accounts for only 13–15% of lung cancers in the general population and approximately 3–19% in patients with IPF [24,25,26,27]. More importantly, the diagnosis and treatment plans for unbiopsied patients were carefully determined through a multidisciplinary team consensus, excluding cases with typical radiological features of SCLC (e.g., rapidly progressive, central bulky mass with extensive mediastinal lymphadenopathy). In addition, histological confirmation was similarly frequent in the two groups (61.1% vs. 58.3%; SMD = 0.057), so any diagnostic misclassification is unlikely to have differed between groups.

Second, radiation dosimetric parameters and treatment techniques were not analyzed in detail. Quantitative dose–volume parameters strongly associated with RP—particularly mean lung dose (MLD), V5, and V20—were not included, and only total radiation dose was evaluated [10,12,14,28]. These quantitative dose–volume parameters were not available in the institutional data warehouse from which this cohort was assembled. Although BED_3_ was balanced between groups and the association was robust to alternative dose specifications, these dose measures do not capture normal lung exposure. Moreover, RP incidence is known to vary with radiation technique, which was not captured in our dataset, and analyses within the ≤2.5 and >2.5 to <6 Gy per fraction categories were not feasible because of small numbers [12,29].

Third, the precise quantitative evaluation of baseline pulmonary fibrosis was limited. Although patients were classified with UIP or probable UIP based on imaging reports, the quantitative extent of fibrotic lesions and the area of honeycombing on chest CT were not measured. Despite being a strong predictor of acute exacerbation and pulmonary toxicity, the extent of baseline fibrosis could not be stratified by severity in our analysis [15]. The GAP (gender, age and physiology) index was also not available.

Fourth, RP was ascertained retrospectively by non-blinded investigators under routine rather than protocol-based imaging surveillance, the frequency of which could not be compared between groups; more frequent imaging in patients with IPF could have increased the detection of asymptomatic RP, although the association was at least as strong for symptomatic grade ≥ 2 RP. Infection within the irradiated field also could not be reliably distinguished from RP. Fifth, residual imbalance remained in several variables that were not matching covariates, including prior chemotherapy, ICI exposure, hypertension, prior extrapulmonary malignancy, tumor location and pulmonary function indices other than FVC. Although ICI exposure was adjusted for and the association was robust to additional adjustment for tumor location, DLCO or FEV1/FVC, residual confounding, particularly by lung dose–volume parameters and the extent of fibrosis, cannot be excluded, and the estimates should be regarded as adjusted associations rather than precise measures of a causal effect.

Sixth, a single completed dataset was analyzed rather than estimates pooled across imputations, so the uncertainty introduced by imputing percent-predicted FVC is not reflected in the confidence intervals. Imputation was, however, confined to one matching covariate that was missing in 12.2% of patients, and a complete-case analysis restricted to the 172 patients in whom FVC had been measured gave a similar estimate (adjusted OR 3.98 vs. 3.55; Table S2).

Finally, as a single-center study of a selected radiotherapy-treated cohort with a median follow-up of 27 months, the generalizability of our findings requires confirmation in multicenter cohorts.

## 5. Conclusions

In this RT-treated cohort, comorbid IPF was independently associated with a higher risk of RP at every severity threshold and with shorter overall survival. Because the cumulative incidence of RP diverged within the first months after RT, close multidisciplinary monitoring during this early period is warranted in patients with IPF. Grade 5 RP occurred in 2 of 72 patients with IPF (2.8%) and in none without IPF; given the small number of events, this frequency is imprecise, and because this study included no non-irradiated comparator or tumor-control endpoint, it cannot address the net clinical benefit of RT in this population. Prospective studies incorporating dose–volume parameters and a quantitative assessment of fibrosis are needed to refine risk stratification and to evaluate preventive strategies.

## Figures and Tables

**Table 1:** Comparison of baseline characteristics between patients with NSCLC, according to comorbid IPF.

Characteristic	IPF (*n* = 72)	No IPF (*n* = 124)	*p*-Value	SMD
Age, year	72.39 ± 7.37	72.48 ± 9.65	0.943	0.011
Male sex (%)	65.0 (90.3)	111.1 (89.6)	0.875	0.023
BMI, kg/m^2^	24.47 ± 3.31	24.43 ± 3.49	0.938	0.012
Smoking history (%)			0.995	0.015
Never smoker	7.0 (9.7)	12.1 (9.7)		
Ex-smoker	42.0 (58.3)	73.2 (59.0)		
Current smoker	23.0 (31.9)	38.8 (31.2)		
Smoking pack-years	39.38 ± 23.33	34.40 ± 19.85	0.129	0.230
Past history (%)				
Hypertension	32.0 (44.4)	74.1 (59.7)	0.043	0.309
Diabetes mellitus	31.0 (43.1)	43.1 (34.7)	0.263	0.172
COPD	21.0 (29.2)	47.4 (38.2)	0.212	0.192
Asthma	4.0 (5.6)	10.3 (8.3)	0.482	0.109
Old tuberculosis	11.0 (15.3)	9.5 (7.6)	0.096	0.242
Stroke	3.0 (4.2)	12.1 (9.7)	0.171	0.220
Cardiovascular disease	21.0 (29.2)	53.4 (43.1)	0.060	0.292
Rheumatic disorder	0.0 (0.0)	2.6 (2.1)	0.188	0.206
Prior extrapulmonary malignancy †	3.0 (4.2)	20.7 (16.7)	0.011	0.418
Lung cancer-related treatment (%)				
Surgery	8.0 (11.1)	18.1 (14.6)	0.504	0.104
Chemotherapy	5.0 (6.9)	27.6 (22.2)	0.006	0.443
ICI	4.0 (5.6)	24.1 (19.4)	0.009	0.430
CCRT	13.0 (18.1)	31.9 (25.7)	0.231	0.186
ECOG performance status (%)			0.805	0.177
0	11.0 (15.3)	18.9 (15.3)		
1	60.0 (83.3)	100.8 (81.2)		
2	1.0 (1.4)	2.6 (2.1)		
3	0.0 (0.0)	1.7 (1.4)		
Pulmonary function test				
FVC, % predicted	81.7 ± 16.4	81.5 ± 16.0	0.921	0.015
FEV_1_, % predicted	87.7 ± 17.0	76.8 ± 20.0	<0.001	0.587
FEV_1_/FVC	74.3 ± 11.0	65.5 ± 13.3	<0.001	0.723
DLco, % predicted	57.6 ± 16.1	74.4 ± 21.3	<0.001	0.891
Biopsy performed (%)	44.0 (61.1)	72.3 (58.3)	0.710	0.057
Pathological type (%)			0.297	0.320
Not confirmed	28.0 (38.9)	51.7 (41.7)		
Adenocarcinoma	17.0 (23.6)	35.3 (28.5)		
Squamous cell carcinoma	27.0 (37.5)	33.6 (27.1)		
Other	0.0 (0.0)	3.4 (2.8)		
TNM stage (%)			0.915	0.108
I	49.0 (68.1)	84.4 (68.1)		
II	5.0 (6.9)	6.9 (5.6)		
III	15.0 (20.8)	25.0 (20.1)		
IV	3.0 (4.2)	7.8 (6.3)		
Tumor size category (%)			0.541	0.215
T1	42.0 (58.3)	81.8 (66.0)		
T2	17.0 (23.6)	18.9 (15.3)		
T3	6.0 (8.3)	11.2 (9.0)		
T4	7.0 (9.7)	12.1 (9.7)		
Tumor location (%)			0.174	0.385
Right upper lobe	16.0 (22.2)	40.5 (32.6)		
Right middle lobe	2.0 (2.8)	6.9 (5.6)		
Right lower lobe	25.0 (34.7)	25.0 (20.1)		
Left upper lobe	17.0 (23.6)	33.6 (27.1)		
Left lower lobe	12.0 (16.7)	18.1 (14.6)		
Purpose of radiotherapy (%)			0.678	0.064
Definitive	64.0 (88.9)	107.6 (86.8)		
Palliative	8.0 (11.1)	16.4 (13.2)		
Radiotherapy total dose, Gy	53.1 ± 9.4	52.5 ± 11.3	0.717	0.054
Dose per fraction, Gy	9.0 ± 4.3	10.0 ± 6.5	0.206	0.177
Number of fractions	9.3 ± 8.8	10.5 ± 10.4	0.399	0.126
BED_3_, Gy ‡	211.0 ± 82.6	215.2 ± 93.3	0.750	0.047
Fractionation (%)			0.644	0.142
≤2.5 Gy per fraction	12.0 (16.7)	25.8 (20.8)		
>2.5 to <6 Gy per fraction	5.0 (6.9)	11.2 (9.0)		
≥6 Gy per fraction	55.0 (76.4)	87.0 (70.1)		
Baseline KL-6, U/mL, median (IQR)§	668 (478–1014)			
ILD treatment (%)	42 (58.3)			
Antifibrotics	37 (51.4)			
Immunosuppressant	5 (6.9)			

Plus–minus values are means ±SD. Weighted values are shown after propensity score weighting; percentages may not total 100 because of rounding. A *P*-value of less than 0.05 was considered to indicate statistical significance. Balance between groups was additionally assessed with the standardized mean difference (SMD), with a value below 0.1 generally considered to indicate good balance. NSCLC, non-small cell lung cancer; IPF, idiopathic pulmonary fibrosis; BMI, body mass index; COPD, chronic obstructive pulmonary disease; ICI, immune checkpoint inhibitor; CCRT, concurrent chemoradiotherapy; ECOG, Eastern Cooperative Oncology Group; FVC, forced vital capacity; FEV1, forced expiratory volume in 1 s; DLco, diffusing capacity of the lung for carbon monoxide; TNM, tumor–node–metastasis; KL-6, Krebs von den Lungen-6. † Defined as a history of non-lung cancer diagnosed >5 years prior to the current NSCLC diagnosis. ‡ BED_3_, biologically effective dose (α/β = 3 Gy), calculated as *n* × d × (1 + d/3), where *n* is the number of fractions, and d is the dose per fraction (Gy). §KL-6 was measured in a subgroup of 35 patients with IPF.

**Table 2:** Incidence, risk and time to onset of radiation pneumonitis (RP) according to comorbid IPF.

Variable	IPF-Comorbid Group (*n* = 72)	Non-IPF Group (*n* = 124)	Odds Ratio (95% CI) *	*p*-Value
Radiation Pneumonitis—no. (%)				
Any-grade	40 (55.6)	38 (30.6)	2.75 (1.48–5.11)	0.001
Grade ≥ 2	31 (43.1)	25 (20.2)	2.87 (1.48–5.57)	0.002
Grade ≥ 3	12 (16.7)	7 (5.6)	3.00 (1.07–8.38)	0.036
Grade 5 (fatal) ^†^	2 (2.8)	0 (0)	—	—
Time to Onset (days) ^‡^—median (IQR)				
Median (IQR)	111.5 (62.0–145.5)	126.5 (84.8–150.8)	—	0.060 ^⸸^

IPF, idiopathic pulmonary fibrosis; CI, confidence interval; IQR, interquartile range. * Odds ratios and *p*-values for the risk of radiation pneumonitis were calculated with propensity score-weighted logistic regression. † The odds ratio could not be calculated because there were no grade 5 events in the non-IPF group. ‡ Time to onset was calculated only among patients in whom radiation pneumonitis developed. ⸸ The *p*-value for median time to onset was calculated using the Wilcoxon rank-sum test.

**Table 3:** Univariable and multivariable weighted logistic regression of factors associated with any-grade radiation pneumonitis.

Variable	Univariable Analysis		Multivariable Analysis	
	OR (95% CI)	*p*-Value	aOR (95% CI)	*p*-Value
IPF	2.75 (1.48–5.11)	0.001	3.55 (1.74–7.24)	0.001
Age	1.01 (0.98–1.04)	0.668	1.04 (1.00–1.08)	0.027
Male gender	0.59 (0.24–1.46)	0.255	0.46 (0.18–1.15)	0.096
ECOG PS	1.09 (0.56–2.12)	0.804		
Smoker				
Current (vs. never)	1.30 (0.45–3.79)	0.626		
Ex-smoker vs. never	1.24 (0.46–3.34)	0.673		
Smoking pack year	1.00 (0.99–1.01)	0.881		
Prior extrapulmonary malignancy †	1.78 (0.73–4.37)	0.205		
Pathology type				
Adeno vs. not confirmed	1.42 (0.67–3.01)	0.353		
SCC vs. not confirmed	1.45 (0.71–2.93)	0.304		
Others vs. not confirmed	0.61 (0.05–7.52)	0.697		
Lung cancer-related treatment				
Surgery	2.00 (0.83–4.82)	0.123	3.14 (1.13–8.71)	0.029
ICI	1.84 (0.81–4.21)	0.146	1.35 (0.40–4.60)	0.629
CCRT	2.38 (1.17–4.84)	0.017	2.33 (0.81–6.70)	0.115
Tumor size	1.40 (1.04–1.88)	0.025	1.31 (0.86–2.01)	0.210
FVC, predicted (%)	1.01 (0.99–1.03)	0.473		
RT total dose (Gy)	1.03 (1.00–1.07)	0.051	1.03 (1.00–1.07)	0.041

Odds ratios (ORs) and adjusted odds ratios (aORs) are shown with 95% confidence intervals (CIs). IPF, idiopathic pulmonary fibrosis; ECOG PS, Eastern Cooperative Oncology Group performance status; ICI, immune checkpoint inhibitor; CCRT, concurrent chemoradiotherapy; TNM, tumor–node–metastasis; FVC, forced vital capacity; RT, radiotherapy; RP, radiation pneumonitis. † Defined as a history of non-lung cancer diagnosed >5 years prior to the current NSCLC diagnosis.

**Table 4:** Univariable and multivariable propensity score-weighted Cox proportional hazards regression of factors associated with overall survival.

Variable	Univariable Analysis		Multivariable Analysis	
	HR (95% CI)	*p*-Value	aHR (95% CI)	*p*-Value
IPF	1.87 (1.12–3.14)	0.018	2.51 (1.30–4.84)	0.006
Age	0.99 (0.96–1.01)	0.235	1.02 (0.99–1.06)	0.222
Male gender	2.12 (0.64–7.05)	0.218	0.55 (0.13–2.30)	0.415
ECOG PS	1.49 (1.10–2.02)	0.009	1.47 (0.96–2.23)	0.076
Smoker				
Current (vs. never)	7.36 (0.96–56.49)	0.055	3.93 (0.41–37.85)	0.237
Ex-smoker vs. never	7.08 (0.93–53.68)	0.058	4.36 (0.48–39.43)	0.190
Smoking pack year	1.01 (1.00–1.02)	0.026	1.01 (0.99–1.02)	0.257
Prior extrapulmonary malignancy †	0.80 (0.34–1.89)	0.606		
Pathology type				
Adeno vs. not confirmed	4.33 (1.86–10.07)	< 0.001	2.96 (1.28–6.85)	0.011
SCC vs. not confirmed	5.23 (2.25–12.14)	< 0.001	2.26 (0.91–5.62)	0.079
Others vs. not confirmed	18.42 (5.89–57.61)	< 0.001	22.75 (6.41–80.78)	< 0.001
Lung cancer-related treatment				
Surgery	0.55 (0.27–1.12)	0.099	0.47 (0.21–1.03)	0.058
ICI	1.84 (0.98–3.46)	0.060	0.70 (0.24–2.01)	0.502
CCRT	2.60 (1.52–4.45)	< 0.001	2.15 (0.78–5.88)	0.137
TNM staging	1.66 (1.32–2.08)	<0.001	1.51 (1.08–2.11)	0.016
FVC, predicted (%)	0.97 (0.96–0.99)	0.003	0.97 (0.95–0.99)	0.015
RT total dose (Gy)	1.01 (0.97–1.05)	0.612		

Hazard ratios (HRs) and adjusted hazard ratios (aHRs) are shown with 95% confidence intervals (CIs). IPF, idiopathic pulmonary fibrosis; ECOG PS, Eastern Cooperative Oncology Group performance status; ICI, immune checkpoint inhibitor; CCRT, concurrent chemoradiotherapy; TNM, tumor–node–metastasis; FVC, forced vital capacity; RT, radiotherapy. † Defined as a history of non-lung cancer diagnosed >5 years prior to the current NSCLC diagnosis. The proportional hazards assumption was not satisfied for IPF status (*p* = 0.002), prior lung surgery (*p* = 0.005), CCRT (*p* = 0.014) or ICI exposure (*p* = 0.037) (global test, *p* = 0.010); the corresponding aHRs therefore represent time-averaged estimates (see text for IPF).

## Data Availability

The datasets generated and analyzed during the current study are not publicly available due to institutional restrictions regarding patient privacy and confidentiality, but de-identified data are available from the corresponding author upon reasonable request.

## Supplementary Materials

The following supporting information can be downloaded at: https://www.mdpi.com/article/10.3390/biomedicines14102280/s1, Figure S1: Propensity score matching results; Table S1: Kaplan–Meier curve results; Table S2: Sensitivity analyses of the association between comorbid IPF and radiation pneumonitis; Table S3: Association of radiotherapy and pulmonary function variables with radiation pneumonitis; Figure S2: The cumulative incidence of radiation pneumonitis with death as a competing event.

## Abbreviations

The following abbreviations are used in this manuscript:

aHR	Adjusted hazard ratio
aOR	Adjusted odds ratio
ATS/ERS/JRS/ALAT	American Thoracic Society/European Respiratory Society/Japanese Respiratory Society/Latin American Thoracic Association
BED_3_	Biologically effective dose (α/β = 3 Gy)
BMI	Body mass index
CCRT	Concurrent chemoradiotherapy
CI	Confidence interval
COPD	Chronic obstructive pulmonary disease
CT	Computed tomography
CTCAE	Common Terminology Criteria for Adverse Events
CTD-ILD	Connective tissue disease-associated interstitial lung disease
DL_CO_	Diffusing capacity of the lung for carbon monoxide
ECOG	Eastern Cooperative Oncology Group
EMR	Electronic medical record
FEV1	Forced expiratory volume in 1 s
FVC	Forced vital capacity
HR	Hazard ratio
ICI	Immune checkpoint inhibitor
ILA	Interstitial lung abnormality
ILD	Interstitial lung disease
IPF	Idiopathic pulmonary fibrosis
IQR	Interquartile range
IRB	Institutional review board
KL-6	Krebs von den Lungen-6
LCC	Large cell carcinoma
MICE-PMM	Multiple imputation by chained equations with predictive mean matching
MLD	Mean lung dose
NSCLC	Non-small cell lung cancer
NSIP	Non-specific interstitial pneumonia
OR	Odds ratio
OS	Overall survival
PS	Performance status
PSM	Propensity score matching
RP	Radiation pneumonitis
RT	Radiotherapy
SBRT	Stereotactic body radiotherapy
SCC	Squamous cell carcinoma
SCLC	Small cell lung cancer
SCRAP	Severance Clinical Research Analysis Portal
SMD	Standardized mean difference
TNM	Tumor–node–metastasis
UIP	Usual interstitial pneumonia
V5	Percentage of lung volume receiving ≥5 Gy
V20	Percentage of lung volume receiving ≥20 Gy
