# Supplementary material for: The Impact of Idiopathic Pulmonary Fibrosis on the Incidence and Severity of Radiation Pneumonitis in Non-Small Cell Lung Cancer Patients Following Radiotherapy
Source: Biomedicines. 2026 Oct 8;14(10):2280. doi: 10.3390/biomedicines14102280 (PMC13701241; doi:10.3390/biomedicines14102280)
Supplement: Supplementary file 1 [file biomedicines-14-02280-s001.zip › Table S3. Association of radiotherapy and pulmonary function variables with any-grade radiation pneumonitis.pdf]

Table S3. Association of radiotherapy and pulmonary function variables with any-grade radiation pneumonitis.

**Variable**	**RP, no./total (%)**	**Adjusted OR (95% CI)**	***p* Value**
Radiation dose and fractionation			
BED_3, per Gy*	—	0.999 (0.994–1.004)	0.674
Fractionation category†			
≤2.5 Gy per fraction	22/36 (61.1)	Reference	—
>2.5 to <6 Gy per fraction	9/18 (50.0)	0.41 (0.11–1.54)	0.185
≥6 Gy per fraction	47/142 (33.1)	0.12 (0.02–0.59)	0.010
Tumor location‡			
Upper lobe	39/109 (35.8)	Reference	—
Lower lobe	36/77 (46.8)	1.41 (0.70–2.84)	0.327
Middle lobe	3/10 (30.0)	0.81 (0.26–2.49)	0.714
Pulmonary function§			
DL_{CO}, per 1% predicted (n = 134)	—	1.01 (0.99–1.03)	0.370
FEV_1/FVC, per 1% (n = 172)	—	0.98 (0.95–1.02)	0.301

Each estimate is taken from the corresponding model in Table S2: survey-weighted logistic regression for any-grade RP in the propensity score-matched cohort, weighted by the matching weights and adjusted for comorbid IPF, age, sex, prior lung surgery, immune checkpoint inhibitor exposure, concurrent chemoradiotherapy and tumor size. Numbers of patients with RP are crude (unweighted).

* From the model in which total radiation dose was replaced by BED_3 (α/β = 3 Gy).

† From the model in which total radiation dose was replaced by fractionation category.

‡ From the primary model (which includes total radiation dose) with tumor location added; the unadjusted odds ratios were 1.60 (95% CI 0.86–2.96, p = 0.138) for lower- and 0.85 (0.21–3.55, p = 0.827) for middle-lobe location.

§ From the primary model with DL_{CO} or FEV_1/FVC added, among patients in whom the test had been performed; each variable was added separately.

RP, radiation pneumonitis; IPF, idiopathic pulmonary fibrosis; OR, odds ratio; CI, confidence interval; BED_3, biologically effective dose (α/β = 3 Gy); DL_{CO}, diffusing capacity of the lung for carbon monoxide; FEV_1, forced expiratory volume in 1 second; FVC, forced vital capacity.
