# Supplementary material for: The Impact of Idiopathic Pulmonary Fibrosis on the Incidence and Severity of Radiation Pneumonitis in Non-Small Cell Lung Cancer Patients Following Radiotherapy
Source: Biomedicines. 2026 Oct 8;14(10):2280. doi: 10.3390/biomedicines14102280 (PMC13701241; doi:10.3390/biomedicines14102280)
Supplement: Supplementary file 1 [file biomedicines-14-02280-s001.zip › Figure S1. PSM results.pdf]

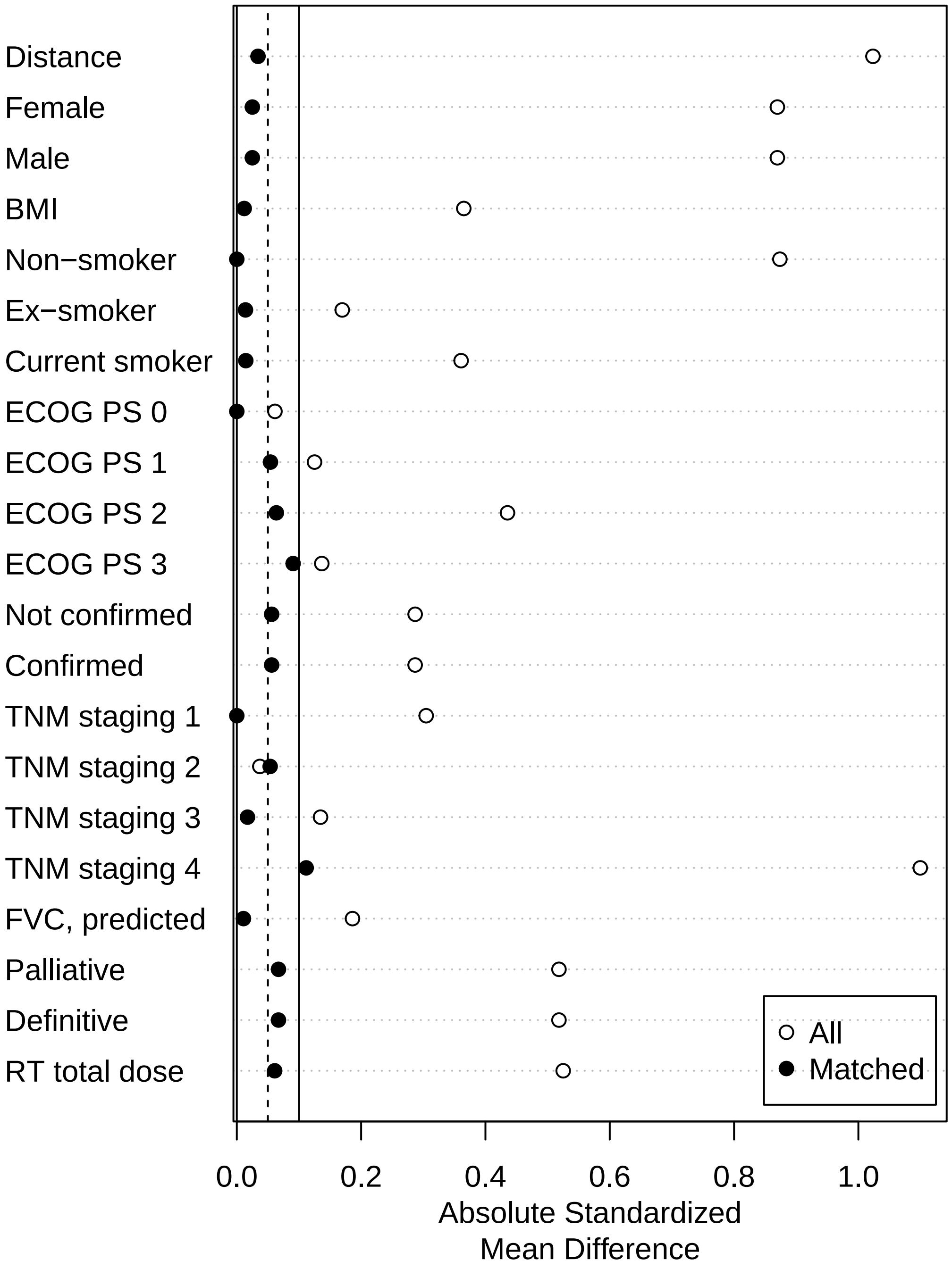

Figure S1. Propensity Score Matching Results.

BMI, body-mass index; ECOG PS, Eastern Cooperative Oncology Group performance status; TNM, tumor–node–metastasis; FVC, forced vital capacity; RT, radiotherapy.
