# Supplementary material for: The Impact of Idiopathic Pulmonary Fibrosis on the Incidence and Severity of Radiation Pneumonitis in Non-Small Cell Lung Cancer Patients Following Radiotherapy
Source: Biomedicines. 2026 Oct 8;14(10):2280. doi: 10.3390/biomedicines14102280 (PMC13701241; doi:10.3390/biomedicines14102280)

Figure S2. Cumulative incidence of radiation pneumonitis

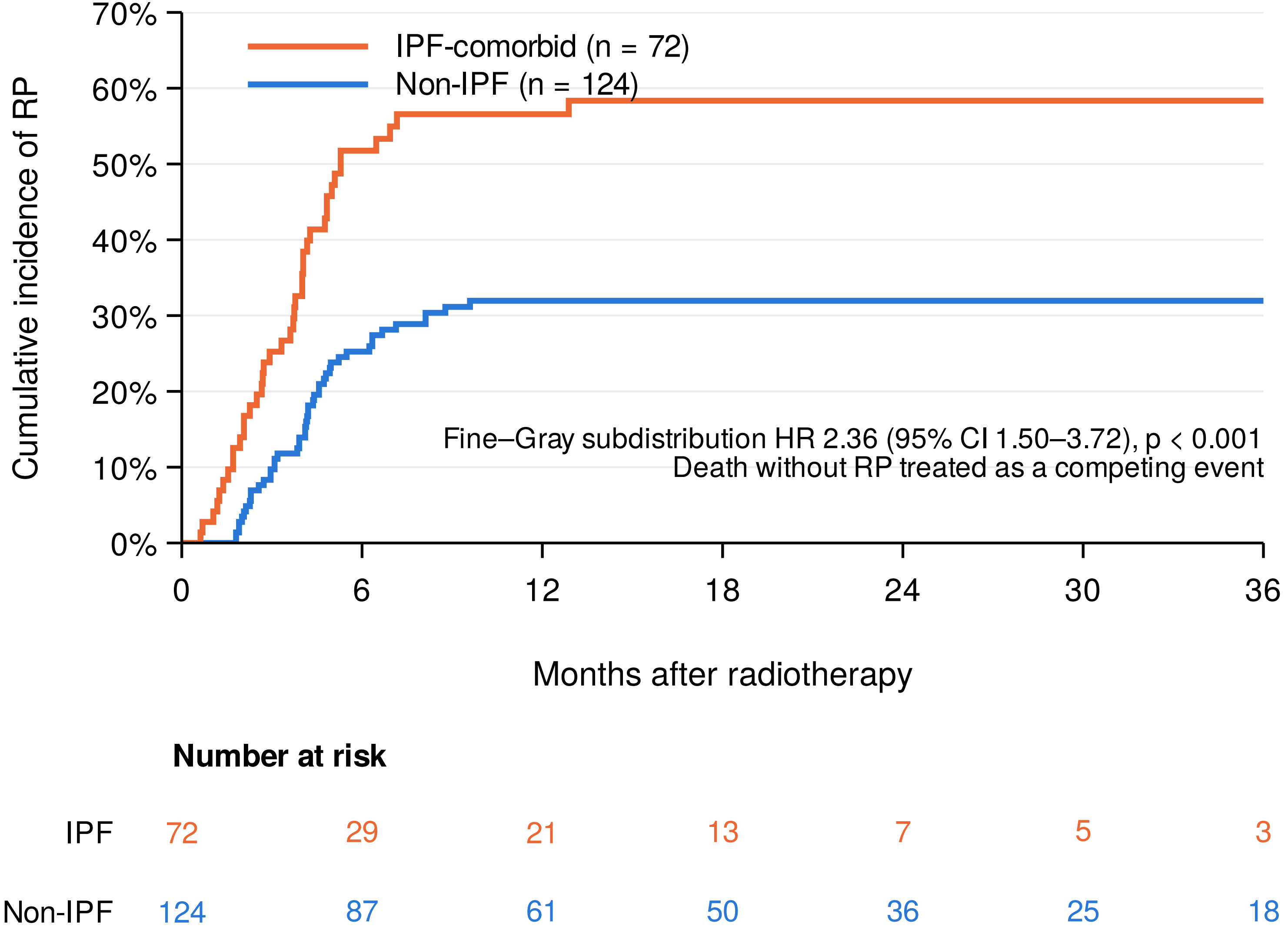

Supplement: Supplementary file 1 [file biomedicines-14-02280-s001.zip › Figure S2. Cumulative incidence of RP.pdf]
