# Supplementary material for: The Impact of Idiopathic Pulmonary Fibrosis on the Incidence and Severity of Radiation Pneumonitis in Non-Small Cell Lung Cancer Patients Following Radiotherapy
Source: Biomedicines. 2026 Oct 8;14(10):2280. doi: 10.3390/biomedicines14102280 (PMC13701241; doi:10.3390/biomedicines14102280)
Supplement: Supplementary file 1 [file biomedicines-14-02280-s001.zip › Table S1. Kaplan-Meier Result.pdf]

Table S1. Kaplan-Meier curve results

Overall Survival Rate				
**Characteristic**	**6 Month OS**	**12 Month OS**	**36 Month OS**	**p-value**[1]
group				0.011
Non-IPF	99% (97%, 100%)	95% (91%, 99%)	68% (57%, 81%)	
IPF	90% (83%, 97%)	82% (73%, 92%)	44% (32%, 61%)	

[1]Log-rank test
