# Supplementary material for: The Impact of Idiopathic Pulmonary Fibrosis on the Incidence and Severity of Radiation Pneumonitis in Non-Small Cell Lung Cancer Patients Following Radiotherapy
Source: Biomedicines. 2026 Oct 8;14(10):2280. doi: 10.3390/biomedicines14102280 (PMC13701241; doi:10.3390/biomedicines14102280)
Supplement: Supplementary file 1 [file biomedicines-14-02280-s001.zip › Table S2. Sensitivity analyses of the association between comorbid IPF and radiation pneumonitis.pdf]

Table S2. Sensitivity analyses of the association between comorbid IPF and radiation pneumonitis.

**Analysis**	**n**	**Adjusted OR or HR (95% CI)**	***p* Value**
Severity of radiation pneumonitis (primary model)*			
Any-grade RP (78 events)	196	3.55 (1.74–7.24)	0.001
Grade ≥2 RP (56 events)	196	3.79 (1.77–8.09)	0.001
Grade ≥3 RP (19 events)†	196	3.03 (1.07–8.60)	0.037
Alternative specification of radiation dose (any-grade RP)‡			
Total dose replaced by BED_3	196	3.43 (1.70–6.90)	0.001
Total dose replaced by fractionation category	196	3.74 (1.81–7.73)	<0.001
Additional adjustment (any-grade RP)‡			
Primary model + tumor location	196	3.33 (1.61–6.92)	0.001
Patients with measured DL_{CO}: primary model	134	4.93 (2.09–11.65)	<0.001
Patients with measured DL_{CO}: primary model + DL_{CO}	134	5.71 (2.32–14.02)	<0.001
Patients with measured spirometry: primary model	172	3.98 (1.87–8.49)	<0.001
Patients with measured spirometry: primary model + FEV_1/FVC	172	4.65 (2.03–10.62)	<0.001
Restricted populations (any-grade RP)			
Patients treated with ≥6 Gy per fraction§	142	3.32 (1.45–7.62)	0.005
IPF with a definite UIP pattern and matched controls‖	157	3.96 (1.74–9.03)	0.001
Death without RP as a competing event (hazard ratios)¶			
Fine–Gray subdistribution hazard model	196	2.46 (1.55–3.93)	<0.001
Cause-specific hazard model	196	2.60 (1.65–4.12)	<0.001

The outcome was any-grade radiation pneumonitis (RP) unless stated otherwise. All models were fitted in the propensity score-matched cohort and weighted by the matching weights; estimates are adjusted odds ratios (ORs) for comorbid IPF from survey-weighted logistic regression, except for the competing-risk analyses, which are hazard ratios (HRs). The associations of the radiotherapy and pulmonary function variables with RP in these models are shown in Table S3.

Unadjusted estimates for comorbid IPF are reported in Table 2 for the whole matched cohort (any-grade RP 2.75, 95% CI 1.48–5.11; grade ≥2 RP 2.87, 1.48–5.57; grade ≥3 RP 3.00, 1.07–8.38) and apply to every analysis fitted in all 196 patients, because the unadjusted model contains comorbid IPF as its only covariate. In the analyses restricted to a subgroup, the unadjusted OR for comorbid IPF was 2.88 (95% CI 1.38–6.03, $p = 0.005$) among patients with measured DL_{CO}, 3.13 (1.62–6.06, $p = 0.001$) among those with measured spirometry, 3.27 (1.54–6.94, $p = 0.002$) among patients treated with ≥6 Gy per fraction and 2.51 (1.26–4.98, $p = 0.009$) in the definite-UIP analysis.

* Adjusted for age, sex, prior lung surgery, immune checkpoint inhibitor exposure, concurrent chemoradiotherapy, tumor size and total radiation dose (Table 3); the same covariates were used in all other models unless stated otherwise.

† Adjusted for age and sex only because of the small number of events; with the full covariate set the adjusted OR was 3.14 (95% CI 1.01–9.79), $p = 0.049$.

‡ BED_3, biologically effective dose ($\alpha/\beta = 3$ Gy), calculated as $n \times d \times (1 + d/3)$, where n is the number of fractions and d the dose per fraction (Gy); fractionation category, ≤2.5 (reference), >2.5 to <6 and ≥6 Gy per fraction; tumor location, upper (reference), lower and middle lobe. DL_{CO} and FEV_1/FVC were added among patients in whom they had been measured, one at a time.

§ RP occurred in 27 of 55 patients (49.1%) with IPF and in 20 of 87 (23.0%) without IPF.

‖ The 58 patients with IPF who had a definite UIP pattern on HRCT, and their matched controls.

¶ Weighted Fine–Gray subdistribution and cause-specific Cox models with robust variance; time from the start of radiotherapy (Figure S2). Without adjustment the subdistribution and cause-specific hazard ratios were 2.36 (95% CI 1.50–3.72) and 2.38 (95% CI 1.51–3.75), respectively (both $p < 0.001$).

IPF, idiopathic pulmonary fibrosis; CI, confidence interval; DL_{CO}, diffusing capacity of the lung for carbon monoxide; FEV_1, forced expiratory volume in 1 second; FVC, forced vital capacity; UIP, usual interstitial pneumonia; HRCT, high-resolution computed tomography.
